# Herbal Neuroprotection Meets Stress-Induced Neuropathology: Bojungikgi-Tang Modulates the Hypothalamic–Pituitary–Adrenal Axis and GABAergic Pathways in Post-Traumatic Stress Disorder

**DOI:** 10.3390/biomedicines13122846

**Published:** 2025-11-21

**Authors:** Mudan Cai, Hee Ra Park, Eun Jin Yang

**Affiliations:** Korean Medicine Science Research Division, Korea Institute of Oriental Medicine (KIOM), 1672 Yuseong-daero, Yuseong-gu, Daejeon 34054, Republic of Korea

**Keywords:** post-traumatic stress disorders, mental disorders, stress, anxiety, cognitive dysfunction, animals, complementary therapies, hippocampus, corticosterone, gamma-aminobutyric acid

## Abstract

**Background**: Post-traumatic stress disorder (PTSD) is a mental disorder that can develop after experiencing or witnessing a traumatic event. Dysfunction of the hypothalamic–pituitary–adrenal (HPA) axis and alterations in neurotransmitters (gamma-aminobutyric acid (GABA) and glutamate) are the main pathologies of PTSD. In particular, altered GABAergic neurotransmission and reduced GABA activity are linked to PTSD. Given the low efficacy and side effects of serotonin reuptake inhibitors—the most common treatment for PTSD—a safer and more effective treatment is urgently needed. Bojungikgi-tang (BJIGT) is well-known herbal prescription in East Asia, which used to boost immunity and to alleviated symptoms such as chronic fatigue, poor appetite, and indigestion. However, its role in PTSD remains largely unexamined. This study aimed to investigate the effects of BJIGT in single-prolonged stress with shock (SPSS)-induced PTSD male mice for 2 weeks. **Methods**: To assess PTSD-like behaviors, we conducted open field, forced swimming, Y-maze, and contextual fear conditioning tests. To investigate the underlying mechanisms, we performed ELISA, Western blot, and immunohistochemistry. **Results**: BJIGT significantly ameliorated PTSD-like behaviors, including emotional and cognitive decline. Additionally, it restored serum corticosterone levels, regulated neuronal functions (c-Fos, DCX, and Prox1), and GABAergic neurotransmission-related factors (vGAT, GAD67, and parvalbumin) in the hippocampus of PTSD mice. Notably, in SPSS-induced PTSD mice, BJIGT effectively ameliorated pathological changes by modulating JNK-CaMKII and Pin1–β-catenin intracellular signaling. **Conclusions**: These findings revealed that BJIGT effectively improved PTSD-like emotional and cognitive decline by regulating the HPA axis and GABAergic neurotransmission in SPSS-induced PTSD mice, thereby promising to be an effective strategy for the treatment of PTSD.

## 1. Introduction

Post-traumatic stress disorder (PTSD) is a mental disorder characterized by common symptoms such as anxiety, hypervigilance, irritability, and depression after a traumatic event [1,2]. Two drugs approved by the Food and Drug Administration (FDA), sertraline (Zoloft) and paroxetine (Paxil), which are selective serotonin reuptake inhibitors (SSRIs), are commonly prescribed for the management of PTSD symptoms. There is no perfect cure for patients with PTSD, particularly for individuals who gradually and repeatedly confront situations and memories [3,4]. Additionally, antipsychotic drugs cause secondary mental problems such as depression, sleep disturbances, and movement disorders. Therefore, treatments for PTSD with fewer adverse effects and greater efficacy are required.

Dysfunction of the hypothalamic–pituitary–adrenal (HPA) axis is the main pathological mechanism in PTSD, and regulation of the level of corticosterone (CORT in animals and cortisol in humans) can attenuate PTSD phenotype development by protecting against persistent hyperarousal and anxiety [5]. In addition, alterations in neurotransmitter homeostasis and neuronal function can contribute to neurological disorders, particularly PTSD [6,7]. For instance, GABA neurotransmitters inhibit the brain function and regulate emotional balance; parvalbumin (PVB) expressed in GABAergic interneurons regulates brain activity and synaptic plasticity, thereby reducing anxiety, fear response, and depression. Several studies have shown a reduced expression of doublecortin (DCX) and Prospero homeobox 1 (Prox1) in the hippocampus of patients with PTSD and that these proteins are associated with neurogenesis, neuronal differentiation, and connectivity [8,9]. These alterations disrupt neuronal function and affect neural circuits involved in fear processing and memory [10].

Bojungikgi-tang (BJIGT, Bu-zhong-yi-qi-tang in Chinese; Hochu-ekki-to in Japanese) is a widely used complementary and alternative medicine (CAM) in East Asia. The main herbs present in BJIGT include Astragali Radix, Glycyrrhizae Radix et Rhizoma, Ginseng Radix, Atractylodis Rhizoma Alba, Citri Unshius Pericarpium, Angelicae Gigan-tis Radix, Cimicifugae Rhizoma, and Bupleuri Radix. BJIGT has been traditionally used to regulate the digestive system and immune system, as well as to alleviate symptoms such as chronic fatigue and poor appetite. BJIGT also improves neurodegenerative disease and atopy-associated gastrointestinal disorder. BJIGT treatment showed neuroprotective effects and improved memory impairment through its antioxidant and anti-inflammatory properties in Alzheimer’s disease [11]. BJIGT protected motor neurons through regulating neuroinflammation, oxidative stress, and autophagy in SOD1^G93A^ mice and a TDP43-expressing cell model [12,13]. Lee et al. also demonstrated that BJIGT improves symptoms of anorexia and atopic dermatitis through modifications in the gut microbiota [14]. Although previous studies have been conducted on BJIGT, its role in PTSD has not yet been examined. Therefore, the aim of this study was to investigate the effects of BJIGT in single-prolonged stress with shock (SPSS)-induced PTSD animal model. The PTSD-like behaviors were examined using the open-field, forced swimming, Y-maze, and contextual fear conditioning tests. Additionally, the possible mechanisms of action of BJIGT on GABAergic neuronal function in PTSD were explored.

## 2. Materials and Methods

### 2.1. Animals

Adult male C57BL/6N mice (7 weeks old) were obtained from Dae Han Biolink (Eumseong County, North Chungcheong Province, Republic of Korea). The mice were housed under specific pathogen-free control conditions and maintained at standard conditions of temperature (21 ± 3 °C) and humidity (50 ± 10%) with a 12 h light/dark cycle and provided ad libitum access to water and food.

### 2.2. Drug Administration

BJIGT was purchased from Hanpoong Pharmaceutical Co Ltd. (Seoul, Republic of Korea). It was dissolved in distilled water and used to treat the PTSD model group. BJIGT (500 or 1000 mg/kg) was continuously administered orally for 14 days at set times. Equal volumes of distilled water were administered to the control and SPSS groups. The dosage was calculated based on the conversion of the dose in adult human subjects according to the literature [15].

### 2.3. SPSS-Induced PTSD Animal Model

PTSD was induced using SPSS as previously described [16,17,18]. Briefly, the mice were subjected to restraint (4 h), forced swimming (20 min), and rest (15 min), and anesthetized using isoflurane. One hour later, the mice were subjected to a foot shock (1 mA, 4 s, twice) in a footshock chamber (Startle & Fear Combined System, Harvard Apparatus, Holliston, MA, USA). Seven days later, additional foot shocks (1 mA, 4 s, four times) were delivered to enhance the hypoarousal response, potentially indicating chronic stress effects. We randomly divided the animals into three groups, and two of the groups underwent the SPSS procedure.

### 2.4. Behavioral Tests

#### 2.4.1. Open Field Test (OFT)

The open field test (OFT) is a validated and widely accepted method for assessing anxiety-like behavior in rodents [19]. The OFT was conducted as previously described [16]. Mice were individually placed in a white Plexiglas box (30 cm × 30 cm × 30 cm) and allowed to move freely and explore for 20 min, during which their behavior was recorded. The recorded videos were analyzed using the EthoVision XT 8.0 system (Noldus Information Technology BV, Wageningen, The Netherlands) to assess various parameters, including the total distance travelled, time spent in central area, and frequency of entries in the center within the defined observation period.

#### 2.4.2. Forced Swimming Test (FST)

The test is one of the most widely used preclinical assays for evaluating depression-like behavior [20]. The FST was performed according to the protocol [21,22]. Mice were acclimated to the testing environment by moving them to the testing area 30 min before the test. Each mouse was individually placed in a glass cylinder (20 cm in diameter, 32 cm in height) filled with water (22–25 °C) to a depth of 15–20 cm, and the total test duration was 6 min. Behaviors were recorded using the EthoVision XT 8.0 system, and the immobility time was analyzed during the final 4 min of the test. The entire procedure was completed within a single day.

#### 2.4.3. Y-Maze Test

The Y-maze test is used to assess short term memory and spatial working memory in mice [23]. The Y-maze test was conducted as previously described [16]. Briefly, the mice were placed in a Y-shaped maze with three arms at 120° angles, and were allowed free access to explore all three arms of the maze for 8 min. A mouse choosing a different arm thrice consecutively, was considered correct, while it was considered invalid, if otherwise (e.g., ABC or BCA, not ABB or ACA). For the analysis, the total number of arm entries and the sequence of entries were recorded by an investigator blinded to group name. The percentage of spontaneous alternations was calculated as follows:

Spontaneous alternation (%) = (number of alternations)/(total arm entries − 2) × 100.(1)

#### 2.4.4. Contextual Fear Conditioning Test (CFCT)

CFCT is used to assess aversive memories associated with a specific environment (conditioning chamber) and were conducted using a Startle & Fear Combined System (Panlab, Barcelona, Spain) [24,25]. One hour after the last administration, mice were re-exposed to the same conditioning chamber for 5 min without receiving foot shocks. Freezing behavior was analyzed for each mouse, with freezing events defined as immobility lasting 2 s or longer, excluding respiratory movements (PACKWIN, Panlab, Barcelona, Spain).

### 2.5. Tissue Preparation

Before anesthesia, the body weight of the mice was measured to determine the level of stress induction. Anesthesia was induced using avertin (Sigma, St. Louis, MO, USA), following which blood samples were collected from the heart. The hippocampus regions were dissected and stored at −70 °C for Western blot analysis or enzyme-linked immunosorbent assay (ELISA). For immunohistochemistry, mice were perfused transcardially with saline, the whole brain was removed immediately and incubated in 4% paraformaldehyde overnight at 4 °C to post-fix. Next, brains were sectioned (thickness of 30 μm) using a cryostat (Leica Microsystems AG, Jena, Germany) and stored in a preservation solution for further analysis.

### 2.6. Corticosterone and GABA ELISA

Blood samples were centrifuged at 3000 rpm at 4 °C for 10 min, and the resulting supernatant were centrifuged again at 12,000 rpm for 10 min. The clear supernatant obtained was collected as serum and stored at −70 °C until further use. For the analysis of serum CORT (Biovision, San Francisco, CA, USA), 10 μL serum was used and assayed following the manufacturer’s instructions. Additionally, a general GABA ELISA kit (Abcam, Cambridge, UK) was used to analyze the GABA levels in the hippocampal regions, according to the manufacturer’s instructions. The optical density (OD) value of each kit was measured using a plate reader (Molecular Devices, San Jose, CA, USA) at absorption wavelengths of 450 nm.

### 2.7. Western Blotting

Western blotting was performed as described previously [26]. Hippocampal tissues were lysed with RIPA buffer (Biosesang, Yongin-si, Republic of Korea) containing phosphatase and protease inhibitors (Thermo Fisher Scientific, Waltham, MA, USA) and centrifuged at 14,000 rpm at 4 °C for 20 min. Sample proteins (20 μg) were separated on Bolt 4–12% Bis-Tris Plus gels (Thermo Fisher Scientific) and electrotransferred onto a polyvinylidene fluoride membrane (Bio-Rad, Hercules, CA, USA). The membranes were incubated with 5% bovine serum albumin for 1 h to block non-specific binding, followed by incubating with primary antibodies at 4 °C overnight. The primary antibodies used were as follows: vGAT (1:1000; Invitrogen, Cambridge, MA, USA); PVB (1:1000; Synaptic Systems, Gottingen, Germany); Tubulin (all 1:1000; Abcam); Phospho-JNK (Thr183/Tyr185) (pJNK), c-Jun N-terminal kinases (JNK), phospho-CaMKII (Thr286), CaMKII, β-catenin (all 1:1000; Cell Signaling Technology, Danvers, MA, USA); Pin1, voltage-gated potassium (Kv4.2) (1:1000; Santa Cruz Biotechnology, Dallas, TX, USA). The next day, the membranes were incubated with the respective secondary antibodies (Santa Cruz Biotechnology) for 1.5 h at room temperature. Protein expression was visualized using a ChemiDoc Imaging System (Bio-Rad) and quantified using ImageJ software (version 1.46j; National Institutes of Health, Bethesda, MD, USA).

### 2.8. Immunohistochemistry (IHC)

Immunohistochemical staining was performed as described previously [26]. The sections were blocked with 5% bovine serum albumin for 1 h at room temperature, then sections were incubated overnight at 4 °C with primary antibodies (c-Fos (1:500; Santa Cruz Biotechnology), DCX (1:500; Santa Cruz Biotechnology), Prox1 (1:1000; Abcam), GAD67 (1:1000; Abcam). The next day, the sections were incubated with appropriate secondary antibodies for 2 h at room temperature. For visualization, the sections were treated with an Avidin-Biotin Complex solution (Vector Laboratories, Burlingame, CA, USA) for 1.5 h and were reacted in the 3,3′-diaminobenzidine peroxidase substrate solution (Vector Laboratories) for maximum 10 min. The tissue sections were mounted on gelatin-coated slides, dehydrated in graded alcohol, and cleared with xylene. Images were captured using a microscope (Olympus BX53; Tokyo, Japan). For c-Fos and GAD67 staining, the number of c-Fos- and GAD67-positive cells was counted blindly following a rigorous protocol to ensure an unbiased analysis. For the immunostaining of DCX and Prox1, the density of the slices was quantified using ImageJ (version 1.46j). The results were quantified from hemispheres of the hippocampus.

### 2.9. Statistical Analysis

All data are expressed as means ± standard errors of the mean (SEM). Statistical analyses were performed using GraphPad Prism software (version 9.0; GraphPad Software, San Diego, CA, USA). Significant differences were determined using a one-way analysis of variance (ANOVA) with Tukey’s multiple comparison test. Statistical significance was set at *p* < 0.05.

## 3. Results

### 3.1. BJIGT Administration Regulates Stress Response

**Figure 1:**
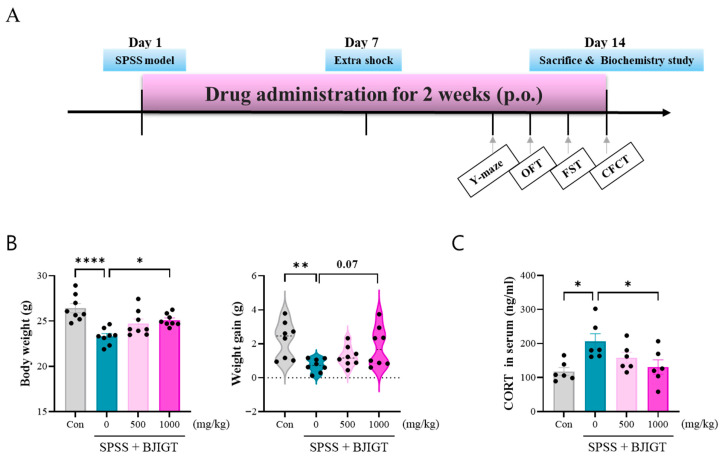
BJIGT administration regulates stress response. (**A**) Experimental design and timeline. All experiments were conducted following this schedule. (**B**) Graph shows the body weight and weight gain before and after drug treatment (*n* = 8/group). (**C**) Quantitative analysis of the levels of serum CORT in each group analyzed using CORT ELISA kit (*n* = 6/group). Con: DW-administered control mice, SPSS: DW-administered SPSS mice, SPSS + BJIGT (500 or 1000 mg/kg): BJIGT-administered SPSS mice. Each filled circle in the graphs represents each mouse. Data were normalized to those of the Con group are presented as mean  ±  standard error of the mean (* *p* < 0.05, ** *p* < 0.01, **** *p* < 0.0001). Con, control; SPSS, single prolonged stress/shock; BJIGT, Bojungikgi-Tang; OFT, open field test; FST, forced swimming test; CFCT, contextual fear conditioning test, CORT, corticosterone, p.o., oral administration.

The experimental schedule is shown in Figure 1. The body weights of mice were significantly lower in the SPSS group than that in the control group, indicating that the mice were stressed, whereas BJIGT administration resulted in a remarkable increase in body weight compared to the SPSS group (Figure 1B). Weight gain was also significantly lower in the SPSS group than that in the control group, whereas BJIGT administration partially restored weight gain compared with the SPSS group, although the difference was not significant (*p* = 0.072). In addition, serum CORT levels, which are stress hormones induced by activation of the HPA axis, were significantly higher in the SPSS group than in the control group. However, BJIGT administration restored CORT levels in the BJIGT group compared to those in the SPSS group (Figure 1C). Overall, these results indicate that BJIGT effectively modulates stress responses (body weight change and HPA axis dysfunction) in a PTSD animal model.

### 3.2. BJIGT Administration Alleviates Emotional and Cognitive Decline

**Figure 2:**
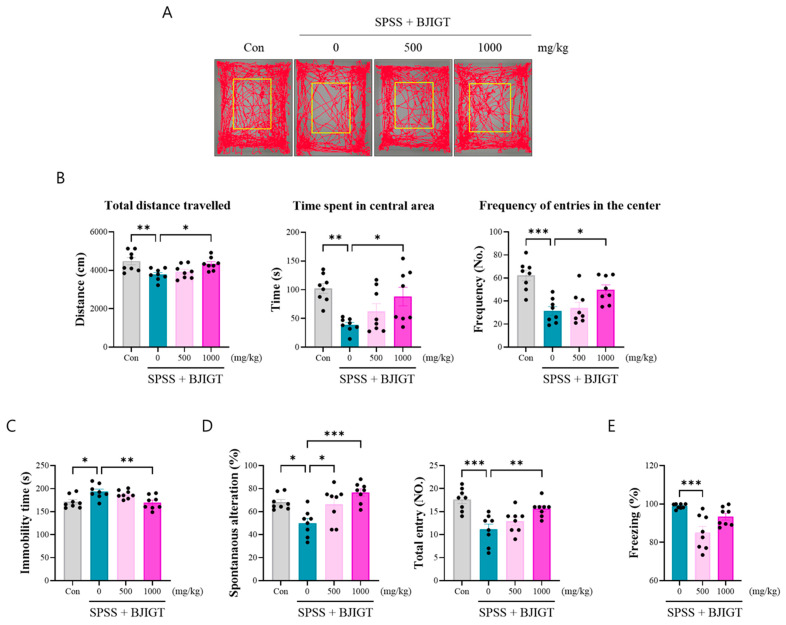
BJIGT administration alleviates emotional and cognitive decline. (**A**) Representative tracking plot of mice in the open field test (OFT). (**B**) Quantitative analysis of the OFT tracking data, including total distance travelled in certain time, time spent in central area, and frequency of entries in the center (*n* = 8/group). (**C**) Quantitative the immobility time of the forced swimming test (*n* = 8/group). (**D**) Quantitative analysis of the Y-maze test, including quantitative spontaneous alteration and total entry (*n* = 8/group). (**E**) Quantitative analysis of the contextual fear condition test, showing the percentage of freezing (*n* = 8/group). Data were normalized to those of the Con group are presented as the mean  ±  standard error of the mean (* *p* < 0.05, ** *p* < 0.01, *** *p* < 0.001).

In this study, we chose SPSS-induced PTSD-like mice, which exhibit PTSD-related phenotypes, such as anxiety, freezing, depression, and cognitive decline. To determine whether BJIGT ameliarated these phenotyes, we investigated the behaviors in the OFT, FST, Y-maze, and FCT. In the OFT, the SPSS group showed dramatically decreased anxiety levels, such as total distance travelled, duration in the center, and frequency of entries in the center (Figure 2A,B). However, BJIGT administration (1000 mg/kg) alleviated those parameters compared to the SPSS group. In the FST, BJIGT administration (1000 mg/kg) significantly decreased the duration of immobility compared to that in the SPSS group, indicating an improvement in depressive emotions (Figure 2C). Furthermore, to analyze cognitive decline, mice were subjected to the Y-maze test to detect hippocampal-dependent short-term memory. BJIGT administration (500 and 1000 mg/kg) significantly increased spontaneous alterations compared with those in the SPSS group (Figure 2D). In addition, the total number of entries was effectively increased by BJIGT administration (1000 mg/kg), indicating an improvement in locomotor activity. Moreover, the CFCT was used to test for hyperarousal, our results showed that BJIGT administration (500 mg/kg) significantly reduced freezing time compared to that in the SPSS group (Figure 2E). These findings indicate that BJIGT administration ameliorated emotional decline, cognitive impairment and hyperarousal through regulation of the HPA axis. Collectively, our findings support the potential of BJIGT as a therapeutic intervention for stress-induced emotional and cognitive deficits.

### 3.3. BJIGT Administration Modulates Neuronal Function

**Figure 3:**
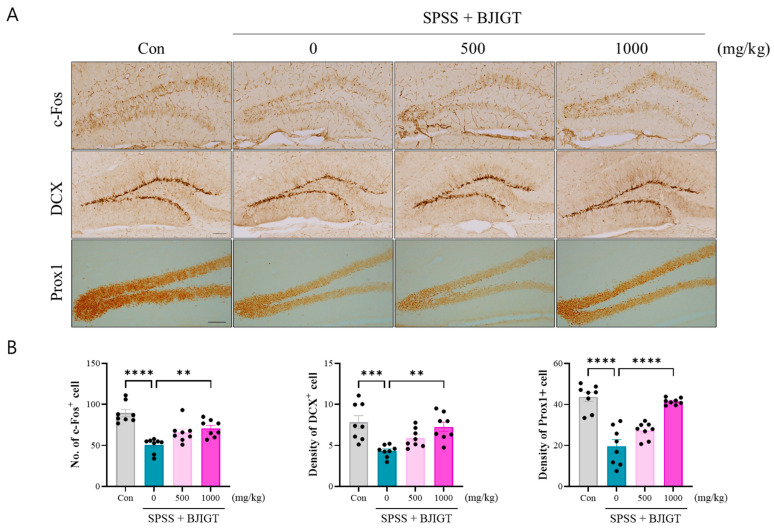
BJIGT administration modulates neuronal function. (**A**) Representative microphotographs of c-Fos, DCX, and Prox1 immunostaining in the dentate gyrus region of the hippocampus in each group. Scale bar = 100 μm. (**B**) Quantification of the number of c-Fos^+^ cells and the densities of DCX^+^ and Prox1+ cells in each group (*n* = 4/group). Data were obtained from both hemispheres of the hippocampus. Data were normalized to those of the Con group are presented as the mean  ±  standard error of the mean (** *p* < 0.01, *** *p* < 0.001, **** *p* < 0.0001).

To investigate brain function, the changes in neuronal activity using c-Fos IHC was performed. The result showed that the number of c-Fos^+^ cells in the dentate gyrus (DG) significantly decreased in the SPSS group. However, BJIGT administration (1000 mg/kg) effectivity regulated the activity of these cells compared to the SPSS group (Figure 3A,B). Additionally, the density of DCX^+^ and Prox1^+^ cells also decreased in the SPSS group, whereas it was restored by BJIGT administration (1000 mg/kg), indicating the restoration of brain development (Figure 3A,B). In addition, dendritic branches were lower in the SPSS group compared to the control group, whereas BJIGT administration (500 and 1000 mg/kg) effectively increased DCX staining, indicating enhanced neurogenesis and improved dendritic organization (Figure 3A). These findings indicate that BJIGT administration effectively restored neuronal activity and neurogenesis in PTSD animal model. Overall, BJIGT appears to support hippocampal plasticity and structural integrity, which may underlie its beneficial effects on cognitive and emotional behaviors.

### 3.4. BJIGT Administration Regulates GABAergic Neurotransmission

**Figure 4:**
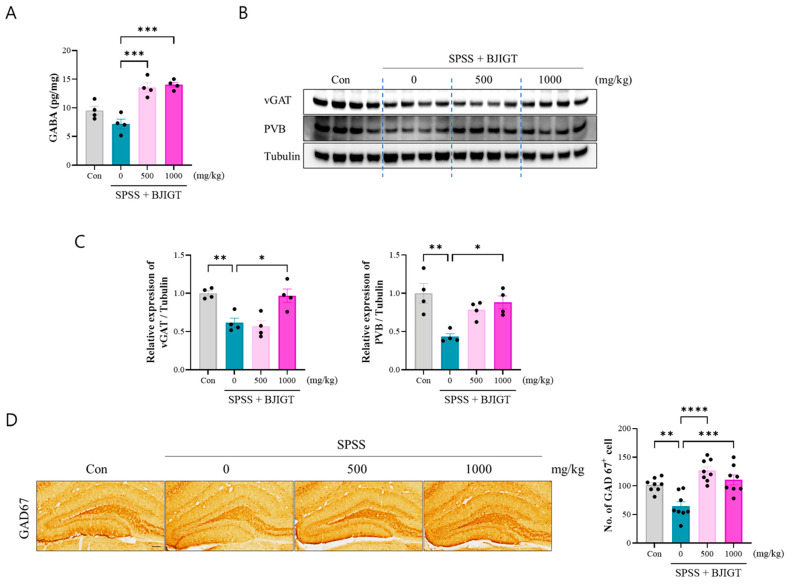
BJIGT administration regulates GABAergic neurotransmission. (**A**) Quantitative analysis of the levels of GABA in the hippocampus of each group analyzed using GABA enzyme-linked immunosorbent assay (*n* = 4/group). (**B**) Representative Western Blot images in each group. (**C**) Quantitative analysis of the bands showing the levels of vGAT/Tubulin and PVB/Tubulin in hippocampus regions of each group. (**D**) Representative microphotographs of GAD67 immunostaining in the hippocampus in each group. Scale bar = 100 μm. Quantification of the number of GAD67^+^ cells in the hippocampus of each group (*n* = 8/group). Data were normalized to those of the Con group are presented as the mean  ±  standard error of the mean (* *p* < 0.05, ** *p* < 0.01, *** *p* < 0.001, **** *p* < 0.0001).

The dysfunction of GABAergic neurons plays a vital role in the development and symptoms of PTSD, especially fear conditioning and extinction. The results of this study showed that BJIGT administration (500 and 1000 mg/kg) increased the level of GABA and it was significantly higher than that observed in the SPSS group (Figure 4A). Furthermore, the expression of vGAT and PVB was significantly decreased in the SPSS group compared to that in the control group, as determined by Western blot, and administration of BJIGT (1000 mg/kg) restored the levels of these proteins (Figure 4B,C). Additionally, IHC revealed that GAD67^+^ cells were significantly decreased in the SPSS group, whereas BJIGT administration (500 and 1000 mg/kg) was significantly increased this parameter compared to the SPSS group, indicating that BJIGT markedly enhances the synthesis of the neurotransmitter GABA (Figure 4D). These results suggest that BJIGT administration effectively restores GABAergic function, which could underlie improvements in fear regulation and other PTSD-related behaviors.

### 3.5. BJIGT Administration Restores Intracellular Signaling Pathways

**Figure 5:**
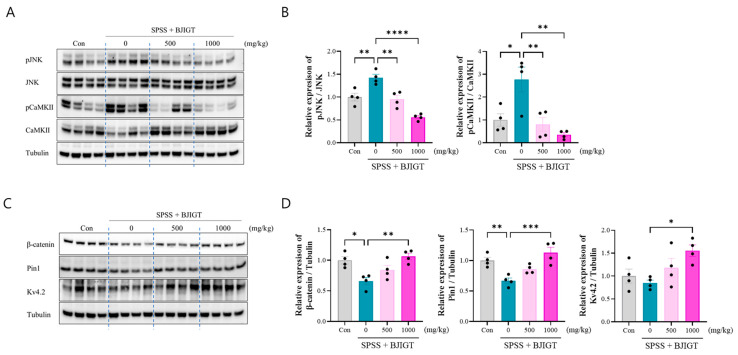
BJIGT administration restores intracellular signaling pathways. (**A**) Representative Western Blot images in each group. (**B**) Quantitative analysis of the bands showing the levels of pJNK/JNK and pCaMKII/CaMKII in the hippocampus regions of each group. (**C**) Representative Western Blot images in each group. (**D**) Quantitative analysis of the bands showing the levels of β-catenin/Tubulin, Pin-1/Tubulin, and Kv4.2/Tubulin in the hippocampus regions of each group. Data were normalized to those of the Con group are presented as the mean  ±  standard error of the mean (* *p* < 0.05, ** *p* < 0.01, *** *p* < 0.001, **** *p* < 0.0001).

To investigate the mechanism of intracellular signaling alterations in PTSD, we analyzed key signaling molecules associated with neuronal excitability, brain function, and potassium channel regulation. It was observed that the levels of pJNK and pCaMKII significantly increased in the SPSS group, whereas BJIGT administration (500 and 1000 mg/kg) was significantly decreased these parameter compared to the SPSS group (Figure 5A,B). Additionally, the levels of β-catenin and Pin-1 were significantly decreased in the SPSS group compared with those in the control group, which was restored upon BJIGT administration (1000 mg/kg) (Figure 5C,D). Voltage-gated potassium (Kv) channels are key regulators of neuronal function. Kv4.2 is involved in the regulation of neuronal excitability, dendritic integration, and cognitive flexibility [27]. The levels of Kv4.2 were slightly lower in the SPSS group compared to the control group (*p* = 0.88). Interestingly, our results showed that BJIGT administration (1000 mg/kg) significantly increased the levels of Kv4.2 compared with those in the SPSS group. Collectively, these results demonstrate that BJIGT modulates intracellular signaling pathways in volved excitability and cognitive function in PTSD animal model, which may contribute to the restoration of neuronal function and the improvement of PTSD-related behavioral deficits.

## 4. Discussion

PTSD is a mental disorder that can develop after experiencing or witnessing a traumatic event, and is characterized by complex symptoms such as fear, anxiety, depression, and insomnia. SPSS is an enhanced PTSD model that produces core PTSD symptoms, including an exaggerated fear response, to better mimic the clinical characteristics of the disorder [17,18,28]. SSRIs are the most common drugs used to treat PTSD. However, SSRIs have lower efficacy and remission, as well as more side effects, for treating patients with PTSD. Therefore, safer and more effective treatments are required. CAM approaches are good candidates because herbal medicines with multi-compound and multi-target characteristics offer potential for development as therapeutics. BJIGT is one of the most well-known herbal medicines used in East Asia. BJIGT restores energy after chronic fatigue or illness and enhances immune function in allergic rhinitis and cancer [29,30]. Recently, Hu et al. quantitatively analyzed 34 components in BJIGT, identifying hesperidin (2478.96 μg/g) as the most abundant, followed by 18β-glycyrrhizic acid (1652.22 μg/g), isoferulic acid (798.30 μg/g), liquiritin (712.79 μg/g), narirutin (641.41 μg/g), ferulic acid (438.01 μg/g), and astragaloside I (232.76 μg/g) [31]. In addition, hesperidin attenuates chronic unpredictable mild stress (CUMS)–induced depression via modulating 5-HT2A receptors [32], and hesperidin also ameliorates Alzheimer’s disease [33]. In particular, total flavonoids from astragalus significantly alleviated depressive behaviors in a CUMS animal model [34]. Additionally, ginsenoside (GS Rg1), the main active ingredient of Radix Ginseng protects against depression-like behaviors in a CUMS model [35]. However, the effects of BJIGT on PTSD have not yet been investigated. Therefore, in this study, we aimed to investigate the effects of BJIGT using matching behavioral tests and examine its molecular mechanisms in an SPSS-induced PTSD animal model.

Dysregulation of the HPA axis is involved in the pathogenesis of mood disorders, including PTSD and depression [36]. The SPSS-induced PTSD model recapitulated stress responses, such as elevated CORT levels and reduced body weight, consistent with our preliminary data and previous studies [26,37]. Our data showed that BJIGT administration restored CORT levels and body weight, indicating inhibition of extremely stressful situations. This finding is consistent with that of Tao et al., who demonstrated that total flavonoids from astragalus, which is one of the components of BJIGT, regulate the levels of CORT and glutamate in the hippocampus in a CUMS model [34]. Dysregulation of the HPA axis disrupts stress homeostasis, leading to alterations in CORT levels, anxiety, depression, hyperarousal, and cognitive decline [5]. In addition, alterations in CORT levels can regulate anxiety and fear memory impairments in the SPSS-induced PTSD rat model [38]. Most studies have demonstrated that chronic treatment with CORT results in anxiety- and depression-like behaviors, as well as PTSD in rodents. Chronic CORT stress leads to glucocorticoid receptor (GR) dysfunction, neuronal plasticity, and the inhibition of oxidative phosphorylation in mitochondria [39]. HPA axis–modulating medications ameliorate PTSD symptoms and lower circulating cortisol levels [40]. Elevated CORT levels in PTSD mice may contribute not only to affective symptoms but also to cognitive impairments. Consistent with this, BJIGT administration ameliorated anxiety- and depression-like behaviors, as assessed by the OFT and FST, and improved cognitive performance and fear memory, as demonstrated in the Y-maze and CFCT. These data suggest that BJIGT administration is beneficial for treating PTSD-like behaviors by alleviating emotional and cognitive decline through the regulation of CORT levels in SPSS-induced PTSD mice.

The hippocampus is a major target of stress mediators and is vulnerable to damage, which can lead to the development of multiple psychiatric disorders, particularly those associated with chronic stress and anxiety disorders [41]. To assess neuronal function, we first measured the expression of c-Fos, an immediate-early gene, is rapidly induced by neuronal activation and plays a significant role in brain function, such as memory formation, stress induction. DCX guides neurons to their correct location (circuit balance) in the developing brain and regulates plasticity in mature neurons. Additionally, DCX promotes dendritic growth and branching, supporting the development of complex neuronal connections. DCX deficiency can lead to reduced dendritic branching and abnormal migration of neuronal progenitor cells [9]. Prox1 is involved in the differentiation of progenitor cells into mature neurons. In the adult DG, Prox1 precursor cells are stimulated by growth factors, physical activity, and enriched environments that contribute to neuronal plasticity and regeneration. Our results showed that c-Fos, DCX, and Prox1 expression were restored by BJIGT administration, indicating increased neuronal function in PTSD. Although neurocircuit assessments were not performed in the present study, these molecular changes may indicate improved hippocampal connectivity and function.

Accumulating evidence indicates that GABAergic system dysfunction contributes to the development of PTSD and its associated symptoms. The inhibitory effect of the GABAergic system is reduced in the hippocampus and prefrontal cortex (PFC) during anxiety and chronic stress [42,43]. In particular, plasma GABA levels are lower in patients with PTSD than that in controls, which could also be considered a possible biomarker for PTSD severity [44]. Our data showed that BJIGT administration significantly increased the GABA levels in SPSS-induced PTSD mice. We further examined GABAergic neurotransmission, in which GAD67 is an enzyme that synthesizes GABA and vGAT transports GABA into vesicles for release. In addition, calcium-binding protein parvalbumin (PVB), which regulates brain activity and synaptic plasticity, is expressed in GABAergic interneurons. Especially, PVB^+^ neurons are important in maintaining neural stability in stressful environments, and their impaired function may weaken the brain’s resilience to stress [45]. Rossetti et al. have suggested that exposure to chronic stress decreases the number of PVB^+^ GABAergic interneurons through an impaired antioxidant response [46]. In addition, restoration of GABAergic neurons contributes to stress resilience by stabilizing inhibitory circuits and protecting against stress-induced synaptic dysfunction [47]. Our data showed that BJIGT administration restored the expression of GAD67, vGAT, and PVB in hippocampus, indicating a regulatory effect on inhibitory GABAergic neurons. Collectively, alterations of GABAergic system have been proven to be one of the key mechanisms of PTSD, characterized by reduced GABA levels, and may serve as a potential therapeutic target.

Hippocampal PVB^+^ GABAergic interneurons regulate emotional memory and stress responses, and their dysfunction can impair dendritic plasticity, contributing to cognitive decline in chronic stress and PTSD [48]. Dysfunction of GABAergic neurons, often driven by altered intracellular Ca^2+^ levels and CaMKII activation, affects neuronal excitability and synaptic plasticity, with Ca^2+^/calmodulin-dependent regulation of GABA_A_ receptors contributing to long-term inhibitory plasticity [49]. In PTSD models, An et al. suggested that suppressing elevated CaMKII activity (Thr286) to normal levels completely rescued PTSD-like behaviors and impairments in long-term depression, particularly the consolidation of fear memory and extinction impairments [50]. Gong et al. demonstrated that the loss of the GABAergic interneuron phenotype, induced by AAV-mediated reduction in GAD67 expression in the hippocampal CA1 region, mediates fear generalization in PTSD rats [51]. Aberrant activation of the JNK pathway via the disruption of GABAergic signaling is associated with cellular stress responses, inflammation, and neuronal dysfunction [52]. Clinical studies and animal experiments have shown that abnormal JNK activation occurs in various psychiatric disorders. Zhou et al. suggested that the activation of JNK modulates depression-like behaviors in mice [53]. Our results obtained in this study support the evidence that intracellular signaling for the activation of CaMKII and JNK is restored by BJIGT administration. In addition, Wnt and β-catenin are related to brain function, synaptic plasticity, processing emotion, and promoting learning and memory [54]. Lv et al. demonstrated that electroacupuncture relieved PTSD-like behaviors by activating the Wnt–β-catenin signaling to modulate impaired hippocampal synaptic plasticity [55]. Several reports demonstrated that Pin1 binds and stabilizes β-catenin in neuronal differentiation; specifically, *Pin1* knockout reduced β-catenin in neuron progenitor cells during brain development [56]. Consistent with our data, Pin1–β-catenin signaling may serve as a therapeutic target for improving cognitive impairment in PTSD. A previous study reported that activity-dependent isomerization of Kv4.2 by Pin1 regulates cognitive flexibility in healthy conditions [27]. Varga et al. demonstrated that CaMKII phosphorylation modulates Kv4.2 expression and directly modulates neuronal excitability by increasing cell-surface expression of A-type K^+^ channels [57]. In addition, Kv4.2 knockout mice control neuronal plasticity and display learning and memory defects [58]. In addition, the expression of Kv 3.1 and Kv4.2 channels is considerably decreased in the hippocampus of rats exposed to chronic mild stress [59]. GABA activates neuronal K^+^ channels, which modulate these channels through intracellular signaling pathways [60]. This supports our present findings that BJIGT administration regulates the expression of Kv4.2 in SPSS mice. In our PTSD model, BJIGT administration involved JNK-CaMKII and Pin 1–β-catenin signaling to modulate intracellular signaling in the hippocampus.

Although BJIGT regulated GABAergic neurons in SPSS-induced PTSD mice, this study has several limitations, including a limited sample size and the use of only male mice; therefore, the results should be interpreted with caution. In addition, the causal links between individual BJIGT components and behavioral effects remain unclear. Future studies should identify the active compounds, investigate region-specific neural mechanisms, and perform clinical and pharmacokinetic evaluations to fully assess BJIGT’s therapeutic potential.

## 5. Conclusions

In summary, BJIGT administration effectively alleviated PTSD-like emotional and cognitive impairment by modulating GABAergic neurons in SPSS-induced PTSD mice. Furthermore, BJIGT restored body weight and CORT levels, improved neuronal function, regulated GABAergic neurotransmission, and normalized intracellular signaling path-ways. These findings highlight the potential of BJIGT as a therapeutic candidate for PTSD. In the future, to better understand the pharmacological basis of BJIGT, we should focus on specific bioactive components responsible for the GABAergic modulation and investigate their region-specific effects on neural circuits involved in stress and emotion regulation. Furthermore, to strengthen the translational potential of BJIGT, we plan to investigate its effects in an additional PTSD model, such as the social defeat stress model and fear extinction paradigm, to more comprehensively validate its efficacy and safety. Although BJIGT showed beneficial effects in a SPSS-induced PTSD model, translating these findings to human therapy requires further clinical and pharmacokinetic evaluation.

## Data Availability

The original contributions presented in this study are included in the article. Further inquiries can be directed to the corresponding authors.

## Abbreviations

The following abbreviations are used in this manuscript:

SSRIs	Selective serotonin reuptake inhibitors
BJIGT	Bojungikgi-tang
BDNF	Brain-derived neurotrophic factor
CAM	Complementary and alternative medicine
CFCT	Contextual fear conditioning test
CORT	Corticosterone
FST	Forced swimming test
GAD67	Glutamate decarboxylase 67
HPA	Hypothalamic–pituitary–adrenal
OFT	Open field test
PTSD	Post-traumatic stress disorder
SPSS	Single-prolonged stress with shock
vGAT	Vesicular GABA transporter
